# Supplementary material for: The Relationship between Immunogenicity and Reactogenicity of Seasonal Influenza Vaccine Using Different Delivery Methods
Source: Vaccines (Basel). 2024 Jul 21;12(7):809. doi: 10.3390/vaccines12070809 (PMC11281354; doi:10.3390/vaccines12070809)
Supplement: Supplementary file 1 [file vaccines-12-00809-s001.zip › vaccines-3090941-supplementary.pdf]

Table of Contents

Supplemental Tables 2

Table S1. Baseline Participant Characteristics 2

Table S2 (A-C). Longitudinal Associations Between Alternative Cut Point Reaction Score and HAI GMT 3

Table S3 (A-C). Reaction Score and HAI GMT by Study Day 6

Table S4 (A-C). Reaction Score and HAI Titer Fold Change by Study Day 9

Table S5. Extreme Reaction Scores and HAI GMT by Study Day 12

Table S6. Extreme Reaction Scores and HAI Titer Fold Change by Study Day 13

Table S7. Reaction Severity and Longitudinal HAI GMT 14

Table S8. Reaction Severity and HAI Titer Fold Change by Study Day 15

Table S9. Reaction Duration and Longitudinal HAI GMT 16

Table S10. Reaction Duration and HAI Titer Fold Change by Study Day 17

Table S11. Longitudinal Associations Between Reaction Score and HAI Seroprotection 18

Table S12. HAI Seroprotection by Vaccine Route and Day* 19

Table S13 (A-C). Longitudinal Associations Between Alternative Cut Point Reaction Score and HAI Seroprotection 20

Table S14. Longitudinal Associations Between Reaction Score and HAI Seroconversion 23

Table S15 (A-C). Reaction Score and HAI Seroconversion by Study Day 24

Table S16 (A-C). Longitudinal Associations Between Alternative Cut Point Reaction Score and HAI Seroconversion 27

Table S17 (A-C). Alternative Cut Point Reaction Score and HAI Seroconversion by Study Day 30

Supplemental Figures 36

Figure S1. Adverse Events by Vaccine Route 36

Figure S2. Select Local Adverse Events by Time and Vaccine Route 37

Figure S3. Reaction Score Univariable Distributions 38

Figure S4. Reaction Score Bivariable Distributions 39

Figure S5. H3N2 HAI Titer by Time and Reaction Score 40

Supplemental Tables

Table S1. Baseline Participant Characteristics.

Characteristic*	IM (n = 25)	MNP (n = 50)	All (n = 75)
Age, year	28.0 (7.0)	26.0 (10.0)	27.0 (9.0)
BMI, kg/m^2	24.8 (6.9)	24.8 (5.3)	24.8 (6.0)
≤ 25	14 (56.0%)	26 (52.0%)	40 (53.3%)
25 – < 30	9 (36.0%)	17 (34.0%)	26 (34.7%)
≥ 30 – ≤ 35	2 (8.0%)	7 (14.0%)	9 (12.0%)
Sex			
Female	11 (44.0%)	24 (48.0%)	35 (46.7%)
Male	14 (56.0%)	26 (52.0%)	40 (53.3%)
Race			
White	12 (48.0%)	25 (50.0%)	37 (49.3%)
Black	8 (32.0%)	16 (32.0%)	24 (32.0%)
Other	5 (20.0%)	9 (18.0%)	14 (18.7%)
Ethnicity			
Not LatinX	22 (88.0%)	48 (96.0%)	70 (93.3%)
LatinX	3 (12.0%)	2 (4.0%)	5 (6.7%)
IIV in prior 2 seasons			
No	17 (68.0%)	34 (68.0%)	51 (68.0%)
Yes	8 (32.0%)	16 (32.0%)	24 (32.0%)
Baseline HAI titer^			
H1N1	80.0 (80.0)	80.0 (140.0)	80.0 (120.0)
H3N2	40.0 (60.0)	40.0 (65.0)	40.0 (60.0)
B	40.0 (40.0)	20.0 (30.0)	40.0 (70.0)
Missing	0	2 (4%)	2 (2.7%)

BMI: body mass index; IIV: inactivated influenza vaccine; HAI: hemagglutination inhibition
*continuous variables: median (IQR); categorical variables: N (%)
^represented as inverse titers

Table S2A. Longitudinal Associations Between Alternative Cut Point Global Reaction Score and HAI GMT.

Reaction Type	Antigen	Study Group	Unadjusted GMR* (95% CI)	p-value	Adjusted GMR*^ (95% CI)	p-value
Global (≥ 3 vs ≤ 2)	H1N1	IM	0.96 (0.51, 1.81)	0.90	0.93 (0.58, 1.51)	0.77
		MNP	0.94 (0.56, 1.58)	0.81	0.91 (0.64, 1.29)	0.59
	H3N2	IM	0.97 (0.57, 1.64)	0.90	0.84 (0.53, 1.33)	0.45
		MNP	0.81 (0.53, 1.26)	0.35	0.78 (0.56, 1.10)	0.15
	B	IM	**1.47 (1.02, 2.12)**	**0.04**	1.16 (0.80, 1.69)	0.42
		MNP	1.04 (0.71, 1.53)	0.82	1.05 (0.76, 1.44)	0.77
Global (≥ 5 vs ≤ 4)	H1N1	IM	0.85 (0.39, 1.87)	0.68	0.81 (0.45, 1.45)	0.47
		MNP	1.03 (0.57, 1.86)	0.91	0.95 (0.64, 1.43)	0.82
	H3N2	IM	0.82 (0.43, 1.58)	0.55	0.73 (0.42, 1.26)	0.25
		MNP	1.00 (0.61, 1.64)	1.0	0.94 (0.64, 1.39)	0.76
	B	IM	0.91 (0.57, 1.45)	0.69	0.69 (0.44, 1.08)	0.10
		MNP	0.91 (0.59, 1.42)	0.68	0.90 (0.63, 1.28)	0.54

HAI: hemagglutination inhibition; GMT: geometric mean titer; GMR: geometric mean titer ratio; CI: confidence interval; IM: intramuscular; MNP: microneedle patch;

*Estimated geometric mean titer ratio for those in the higher score category compared with those in the lower score category

^Adjusted for body mass index category, sex, prior inactivated influenza vaccination, race, ethnicity, and study day

Table S2B. Longitudinal Associations Between Alternative Cut Point Systemic Reaction Score and HAI GMT.

Reaction Type	Antigen	Study Group	Unadjusted GMR* (95% CI)	p-value	Adjusted GMR*^ (95% CI)	p-value
Systemic (≥ 1 vs 0)	H1N1	IM	1.15 (0.60, 2.18)	0.67	1.12 (0.70, 1.81)	0.63
		MNP	0.81 (0.49, 1.35)	0.42	0.8 (0.57, 1.13)	0.21
	H3N2	IM	0.95 (0.56, 1.63)	0.86	0.84 (0.53, 1.32)	0.44
		MNP	1.02 (0.66, 1.56)	0.94	1.03 (0.74, 1.44)	0.87
	B	IM	**1.54 (1.07, 2.22)**	**0.02**	1.22 (0.84, 1.76)	0.29
		MNP	0.87 (0.60, 1.28)	0.48	0.89 (0.66, 1.21)	0.47
Systemic (≥ 2 vs ≤ 1)	H1N1	IM	1.03 (0.55, 1.93)	0.94	1.0 (0.63, 1.59)	0.99
		MNP	1.04 (0.59, 1.84)	0.89	1.12 (0.76, 1.65)	0.56
	H3N2	IM	0.79 (0.47, 1.33)	0.37	0.72 (0.46, 1.11)	0.13
		MNP	0.99 (0.61, 1.60)	0.95	0.99 (0.68, 1.44)	0.94
	B	IM	1.42 (0.99, 2.05)	0.06	1.17 (0.82, 1.67)	0.39
		MNP	0.86 (0.56, 1.33)	0.50	0.87 (0.62, 1.23)	0.43

HAI: hemagglutination inhibition; GMT: geometric mean titer; GMR: geometric mean titer ratio; CI: confidence interval; IM: intramuscular; MNP: microneedle patch;
*Estimated geometric mean titer ratio for those in the higher score category compared with those in the lower score category
^Adjusted for body mass index category, sex, prior inactivated influenza vaccination, race, ethnicity, and study day

Table S2C. Longitudinal Associations Between Alternative Cut Point Local Reaction Score and HAI GMT.

Reaction Type	Antigen	Study Group	Unadjusted GMR* (95% CI)	p-value	Adjusted GMR*^ (95% CI)	p-value
Local (≥ 1 vs 0)	H1N1	IM	1.40 (0.71, 2.73)	0.32	1.34 (0.81, 2.21)	0.25
		MNP	1.18 (0.51, 2.71)	0.69	1.00 (0.57, 1.77)	1.0
	H3N2	IM	1.01 (0.58, 1.77)	0.97	0.92 (0.57, 1.49)	0.73
		MNP	1.00 (0.49, 2.01)	0.99	0.94 (0.54, 1.63)	0.81
	B	IM	1.18 (0.80, 1.76)	0.40	0.95 (0.64, 1.40)	0.79
		MNP	1.19 (0.64, 2.21)	0.59	1.13 (0.68, 1.87)	0.64
Local (≥ 2 vs ≤ 1)	H1N1	IM	1.10 (0.58, 2.09)	0.78	1.02 (0.63, 1.66)	0.92
		MNP	1.19 (0.69, 2.05)	0.53	1.05 (0.72, 1.55)	0.79
	H3N2	IM	1.22 (0.72, 2.08)	0.46	1.05 (0.66, 1.67)	0.83
		MNP	0.79 (0.50, 1.26)	0.32	0.73 (0.50, 1.05)	0.09
	B	IM	1.20 (0.83, 1.75)	0.33	0.92 (0.63, 1.34)	0.66
		MNP	1.30 (0.87, 1.96)	0.20	1.25 (0.89, 1.76)	0.19

HAI: hemagglutination inhibition; GMT: geometric mean titer; GMR: geometric mean titer ratio; CI: confidence interval; IM: intramuscular; MNP: microneedle patch;

*Estimated geometric mean titer ratio for those in the higher score category compared with those in the lower score category

^Adjusted for body mass index category, sex, prior inactivated influenza vaccination, race, ethnicity, and study day

Table S3A. Reaction Score and H1N1 HAI GMT by Study Day.

Reaction Type	Study Group	Day 0 GMR* (95% CI)	p-value	Day 28 GMR* (95% CI)	p-value	Day 180 GMR* (95% CI)	p-value
Unadjusted							
Global	IM	1.0 (0.4, 2.4)	0.99	1.1 (0.5, 2.5)	0.87	1.1 (0.5, 2.7)	0.77
	MNP	1.2 (0.6, 2.3)	0.59	0.7 (0.4, 1.4)	0.36	0.7 (0.4, 1.4)	0.35
Systemic	IM	0.6 (0.2, 1.4)	0.21	0.9 (0.4, 2.1)	0.78	0.8 (0.3, 1.9)	0.58
	MNP	1.5 (0.5, 4.2)	0.48	0.7 (0.2, 2.0)	0.50	0.7 (0.3, 2.1)	0.56
Local	IM	1.0 (0.3, 3.3)	0.94	0.5 (0.1, 1.5)	0.19	0.4 (0.1, 1.3)	0.14
	MNP	1.6 (0.9, 2.9)	0.14	1.5 (0.8, 2.8)	0.18	1.5 (0.8, 2.8)	0.18
Adjusted^							
Global	IM	0.9 (0.4, 2.2)	0.86	1.0 (0.4, 2.3)	0.98	1.0 (0.4, 2.4)	0.92
	MNP	1.2 (0.6, 2.2)	0.64	0.7 (0.4, 1.4)	0.32	0.7 (0.4, 1.4)	0.31
Systemic	IM	0.5 (0.2, 1.2)	0.13	0.8 (0.3, 1.9)	0.59	0.7 (0.3, 1.7)	0.41
	MNP	1.4 (0.5, 3.9)	0.55	0.7 (0.2, 1.9)	0.42	0.7 (0.2, 2.0)	0.48
Local	IM	1.1 (0.3, 3.3)	0.91	0.5 (0.2, 1.5)	0.20	0.4 (0.1, 1.3)	0.14
	MNP	1.6 (0.9, 2.9)	0.14	1.5 (0.8, 2.8)	0.19	1.5 (0.8, 2.8)	0.19

HAI: hemagglutination inhibition; GMT: geometric mean titer; GMR: geometric mean titer ratio; CI: confidence interval; IM: intramuscular; MNP: microneedle patch;
*Estimated geometric mean titer ratio for those in the higher score category compared with those in the lower score category
^Adjusted for body mass index category, sex, prior inactivated influenza vaccination, race, ethnicity, and study day

Table S3B. Reaction Score and H3N2 HAI GMT by Study Day.

Reaction Type	Study Group	Day 0 GMR* (95% CI)	p-value	Day 28 GMR* (95% CI)	p-value	Day 180 GMR* (95% CI)	p-value
Unadjusted							
Global	IM	0.5 (0.2, 1.1)	0.09	1.1 (0.5, 2.4)	0.82	1.0 (0.5, 2.2)	0.93
	MNP	0.8 (0.4, 1.5)	0.53	0.7 (0.4, 1.4)	0.32	0.9 (0.5, 1.6)	0.69
Systemic	IM	**0.4 (0.2, 0.9)**	**0.03**	1.0 (0.5, 2.3)	0.96	0.6 (0.3, 1.4)	0.25
	MNP	1.3 (0.5, 3.6)	0.63	1.5 (0.5, 4.1)	0.47	1.6 (0.6, 4.4)	0.40
Local	IM	**0.3 (0.1, 0.9)**	**0.03**	0.7 (0.2, 1.9)	0.48	0.5 (0.2, 1.3)	0.13
	MNP	0.6 (0.4, 1.2)	0.16	0.7 (0.4, 1.3)	0.29	0.8 (0.4, 1.4)	0.43
Adjusted^							
Global	IM	0.5 (0.2, 1.0)	0.06	1.0 (0.4, 2.2)	0.95	0.9 (0.4, 2.1)	0.85
	MNP	0.8 (0.4, 1.5)	0.45	0.7 (0.4, 1.3)	0.26	0.8 (0.5, 1.6)	0.60
Systemic	IM	**0.4 (0.2, 0.8)**	**0.01**	0.9 (0.4, 1.9)	0.7	0.5 (0.2, 1.2)	0.12
	MNP	1.3 (0.5, 3.5)	0.63	1.4 (0.5, 4.0)	0.47	1.5 (0.6, 4.3)	0.40
Local	IM	**0.3 (0.1, 0.8)**	**0.01**	0.6 (0.2, 1.6)	0.28	0.4 (0.1, 1.1)	0.07
	MNP	0.6 (0.3, 1.0)	0.06	0.6 (0.4, 1.1)	0.13	0.7 (0.4, 1.2)	0.22

HAI: hemagglutination inhibition; GMT: geometric mean titer; GMR: geometric mean titer ratio; CI: confidence interval; IM: intramuscular; MNP: microneedle patch;

*Estimated geometric mean titer ratio for those in the higher score category compared with those in the lower score category

^Adjusted for body mass index category, sex, prior inactivated influenza vaccination, race, ethnicity, and study day

Table S3C. Reaction Score and B HAI GMT by Study Day.

Reaction Type	Study Group	Day 0 GMR* (95% CI)	p-value	Day 28 GMR* (95% CI)	p-value	Day 180 GMR* (95% CI)	p-value
Unadjusted							
Global	IM	1.2 (0.6, 2.3)	0.54	1.2 (0.6, 2.3)	0.58	1.2 (0.6, 2.3)	0.55
	MNP	0.6 (0.3, 1.0)	0.05	1.1 (0.6, 1.9)	0.84	0.9 (0.5, 1.6)	0.66
Systemic	IM	0.9 (0.5, 1.8)	0.83	1.1 (0.6, 2.1)	0.80	1.2 (0.6, 2.4)	0.60
	MNP	1.0 (0.4, 2.5)	0.92	0.9 (0.4, 2.5)	0.90	0.7 (0.2, 1.7)	0.39
Local	IM	1.2 (0.5, 3.0)	0.66	0.8 (0.3, 2.0)	0.68	1.2 (0.5, 2.9)	0.73
	MNP	0.7 (0.4, 1.3)	0.26	1.1 (0.6, 2.0)	0.71	1.1 (0.6, 2.0)	0.65
Adjusted^							
Global	IM	1.0 (0.5, 1.8)	0.89	0.9 (0.5, 1.8)	0.85	0.9 (0.5, 1.8)	0.87
	MNP	**0.6 (0.3, 1.0)**	**0.04**	1.1 (0.6, 1.9)	0.80	0.9 (0.5, 1.5)	0.67
Systemic	IM	0.7 (0.4, 1.4)	0.32	0.8 (0.4, 1.6)	0.59	0.9 (0.5, 1.8)	0.80
	MNP	0.9 (0.4, 2.2)	0.81	0.9 (0.3, 2.2)	0.78	0.6 (0.2, 1.5)	0.29
Local	IM	1.0 (0.4, 2.5)	0.95	0.7 (0.3, 1.7)	0.42	1.0 (0.4, 2.4)	0.98
	MNP	0.7 (0.4, 1.3)	0.29	1.1 (0.7, 2.0)	0.62	1.2 (0.7, 2.0)	0.57

HAI: hemagglutination inhibition; GMT: geometric mean titer; GMR: geometric mean titer ratio; CI: confidence interval; IM: intramuscular; MNP: microneedle patch;

*Estimated geometric mean titer ratio for those in the higher score category compared with those in the lower score category

^Adjusted for body mass index category, sex, prior inactivated influenza vaccination, race, ethnicity, and study day

Table S4A. Reaction Score and H1N1 HAI Titer Fold Change by Study Day.

Reaction Type	Study Group	Day 28 FCR* (95% CI)	p-value	Day 180 FCR* (95% CI)	p-value
Unadjusted					
Global	IM	1.1 (0.3, 3.7)	0.92	1.1 (0.3, 3.9)	0.85
	MNP	0.6 (0.3, 1.4)	0.26	0.6 (0.3, 1.4)	0.25
Systemic	IM	1.5 (0.4, 5.6)	0.51	1.4 (0.4, 5.0)	0.63
	MNP	0.5 (0.1, 1.8)	0.28	0.5 (0.1, 1.9)	0.31
Local	IM	0.4 (0.1, 2.4)	0.34	0.4 (0.1, 2.2)	0.28
	MNP	1.0 (0.4, 2.1)	0.91	1.0 (0.4, 2.1)	0.91
Adjusted^					
Global	IM	1.0 (0.3, 3.1)	0.94	1.0 (0.3, 3.3)	0.99
	MNP	0.7 (0.3, 1.4)	0.30	0.7 (0.3, 1.4)	0.29
Systemic	IM	1.1 (0.3, 4.0)	0.84	1.0 (0.3, 3.5)	1.0
	MNP	0.6 (0.2, 2.0)	0.41	0.6 (0.2, 2.1)	0.45
Local	IM	0.4 (0.1, 2.2)	0.32	0.4 (0.1, 2.0)	0.27
	MNP	1.0 (0.5, 2.2)	0.90	1.0 (0.5, 2.2)	0.90

HAI: hemagglutination inhibition; FCR: fold change ratio; CI: confidence interval; IM: intramuscular; MNP: microneedle patch;
*Estimated fold change ratio for those in the higher score category compared with those in the lower score category
^Adjusted for body mass index category, sex, prior inactivated influenza vaccination, race, ethnicity, and study day

Table S4B. Reaction Score and H3N2 HAI Titer Fold Change by Study Day.

Reaction Type	Study Group	Day 28 FCR* (95% CI)	p-value	Day 180 FCR* (95% CI)	p-value
Unadjusted					
Global	IM	**2.1 (1.1, 4.1)**	**0.03**	**2 (1.0, 3.9)**	**0.04**
	MNP	0.9 (0.5, 1.6)	0.68	1.1 (0.6, 1.9)	0.81
Systemic	IM	**2.4 (1.2, 5.0)**	**0.02**	1.5 (0.7, 3.1)	0.27
	MNP	1.1 (0.4, 2.9)	0.79	1.2 (0.5, 3.1)	0.69
Local	IM	2.2 (0.8, 5.7)	0.12	1.4 (0.5, 3.8)	0.48
	MNP	1.1 (0.6, 1.9)	0.69	1.2 (0.7, 2.1)	0.48
Adjusted^					
Global	IM	**2.0 (1.0, 3.7)**	**0.04**	1.9 (1.0, 3.5)	0.05
	MNP	0.9 (0.6, 1.6)	0.83	1.1 (0.7, 1.9)	0.61
Systemic	IM	1.9 (1.0, 3.8)	0.07	1.2 (0.6, 2.4)	0.64
	MNP	1.3 (0.6, 3.1)	0.49	1.4 (0.6, 3.3)	0.40
Local	IM	2 (0.8, 4.9)	0.15	1.3 (0.5, 3.2)	0.58
	MNP	1.1 (0.7, 1.9)	0.66	1.2 (0.7, 2.0)	0.44

HAI: hemagglutination inhibition; FCR: fold change ratio; CI: confidence interval; IM: intramuscular; MNP: microneedle patch;
*Estimated fold change ratio for those in the higher score category compared with those in the lower score category
^Adjusted for body mass index category, sex, prior inactivated influenza vaccination, race, ethnicity, and study day

Table S4C. Reaction Score and B HAI Titer Fold Change by Study Day.

Reaction Type	Study Group	Day 28 FCR* (95% CI)	p-value	Day 180 FCR* (95% CI)	p-value
Unadjusted					
Global	IM	1.0 (0.4, 2.3)	0.97	1.0 (0.4, 2.4)	0.99
	MNP	1.9 (1.0, 3.6)	0.05	1.6 (0.8, 3.0)	0.16
Systemic	IM	1.2 (0.5, 2.9)	0.73	1.3 (0.5, 3.2)	0.58
	MNP	1.0 (0.3, 2.9)	0.98	0.7 (0.2, 2.0)	0.49
Local	IM	0.7 (0.2, 2.2)	0.52	1.0 (0.3, 3.1)	0.94
	MNP	1.5 (0.8, 2.9)	0.17	1.6 (0.8, 2.9)	0.15
Adjusted^					
Global	IM	0.8 (0.4, 1.6)	0.46	0.8 (0.4, 1.6)	0.48
	MNP	**1.8 (1.1, 3.2)**	**0.03**	1.5 (0.9, 2.6)	0.14
Systemic	IM	0.9 (0.4, 1.9)	0.70	0.9 (0.4, 2.1)	0.88
	MNP	1.1 (0.4, 2.9)	0.79	0.8 (0.3, 2.0)	0.61
Local	IM	0.5 (0.2, 1.5)	0.22	0.7 (0.3, 2.1)	0.58
	MNP	1.3 (0.7, 2.2)	0.39	1.3 (0.7, 2.3)	0.35

HAI: hemagglutination inhibition; FCR: fold change ratio; CI: confidence interval; IM: intramuscular; MNP: microneedle patch;
*Estimated fold change ratio for those in the higher score category compared with those in the lower score category
^Adjusted for body mass index category, sex, prior inactivated influenza vaccination, race, ethnicity, and study day

Table S5. Extreme Reaction Scores and HAI GMT by Study Day.

Reaction Type	Antigen	Study Group	Day 0 GMR* (95% CI)	p-value	Day 28 GMR* (95% CI)	p-value	Day 180 GMR* (95% CI)	p-value
Global	H1N1	IM	0.9 (0.3, 2.8)	0.87	0.8 (0.3, 2.5)	0.74	0.9 (0.3, 2.8)	0.87
		MNP	1.7 (0.5, 5.3)	0.35	1.6 (0.5, 4.8)	0.44	1.4 (0.4, 4.2)	0.60
	H3N2	IM	0.6 (0.2, 1.6)	0.28	1.2 (0.4, 3.4)	0.73	1.1 (0.4, 3.2)	0.84
		MNP	0.5 (0.2, 1.5)	0.24	0.7 (0.3, 1.9)	0.47	0.9 (0.3, 2.4)	0.75
	B	IM	0.9 (0.4, 1.8)	0.68	1.2 (0.5, 2.5)	0.68	1.5 (0.7, 3.2)	0.29
		MNP	0.8 (0.4, 1.8)	0.60	1.0 (0.5, 2.3)	0.95	1.1 (0.5, 2.4)	0.86
Systemic	H1N1	IM	0.5 (0.2, 1.7)	0.30	0.7 (0.2, 2.3)	0.55	0.5 (0.2, 1.6)	0.24
		MNP	1.5 (0.5, 4.5)	0.44	0.8 (0.3, 2.3)	0.63	0.6 (0.2, 1.8)	0.35
	H3N2	IM	0.4 (0.1, 1.3)	0.14	1.2 (0.4, 3.8)	0.76	0.8 (0.2, 2.4)	0.65
		MNP	0.5 (0.2, 1.1)	0.08	0.6 (0.3, 1.4)	0.23	0.7 (0.3, 1.5)	0.38
	B	IM	1.0 (0.4, 2.6)	1.0	1.5 (0.6, 4.0)	0.36	1.5 (0.6, 4.0)	0.36
		MNP	1.2 (0.6, 2.4)	0.69	0.7 (0.4, 1.5)	0.41	0.6 (0.3, 1.3)	0.20
Local	H1N1	IM	**4.4 (1.1, 17.7)**	**0.04**	0.3 (0.1, 1.1)	0.07	0.6 (0.2, 2.6)	0.54
		MNP	1.2 (0.5, 2.9)	0.61	1.2 (0.5, 2.7)	0.73	1.4 (0.6, 3.1)	0.47
	H3N2	IM	**0.2 (0.1, 0.8)**	**0.02**	0.3 (0.1, 1.1)	0.06	**0.2 (0.1, 0.8)**	**0.02**
		MNP	0.5 (0.2, 1.4)	0.17	0.6 (0.2, 1.8)	0.40	0.7 (0.2, 1.8)	0.42
	B	IM	2.0 (0.9, 4.3)	0.08	0.9 (0.4, 2.0)	0.82	1.4 (0.7, 3.1)	0.37
		MNP	0.8 (0.4, 1.9)	0.63	0.9 (0.4, 2.1)	0.87	1.3 (0.5, 2.9)	0.58

HAI: hemagglutination inhibition; GMT: geometric mean titer; GMR: geometric mean titer ratio; CI: confidence interval; IM: intramuscular; MNP: microneedle patch;

*Estimated geometric mean titer ratio for those in the higher score category compared with those in the lower score category

Table S6. Extreme Reaction Scores and HAI Titer Fold Change by Study Day.

Reaction Type	Antigen	Study Group	Day 0 FCR* (95% CI)	p-value	Day 28 FCR * (95% CI)	p-value
Global	H1N1	IM	0.9 (0.2, 5.3)	0.92	1.0 (0.2, 5.9)	1.0
		MNP	0.9 (0.3, 2.8)	0.87	0.8 (0.3, 2.5)	0.68
	H3N2	IM	2.1 (1.0, 4.7)	0.06	2 (0.9, 4.4)	0.09
		MNP	1.3 (0.5, 3.1)	0.61	1.6 (0.6, 3.8)	0.33
	B	IM	1.4 (0.8, 2.4)	0.29	1.7 (1.0, 3.1)	0.06
		MNP	1.3 (0.5, 3.0)	0.59	1.3 (0.5, 3.2)	0.52
Systemic	H1N1	IM	1.3 (0.2, 8.4)	0.78	0.9 (0.1, 6.0)	0.92
		MNP	0.5 (0.2, 1.4)	0.18	0.4 (0.1, 1.1)	0.07
	H3N2	IM	**2.8 (1.2, 6.5)**	**0.02**	1.8 (0.8, 4.2)	0.15
		MNP	1.3 (0.6, 2.6)	0.54	1.4 (0.7, 3.0)	0.34
	B	IM	1.5 (0.7, 3.3)	0.25	1.5 (0.7, 3.3)	0.25
		MNP	0.6 (0.3, 1.5)	0.31	0.5 (0.2, 1.3)	0.17
Local	H1N1	IM	**0.1 (0.0, 0.5)**	**0.01**	0.1 (0.0, 1.2)	0.07
		MNP	0.9 (0.3, 3.1)	0.91	1.1 (0.3, 3.7)	0.88
	H3N2	IM	1.4 (0.5, 4.2)	0.52	1.1 (0.4, 3.2)	0.87
		MNP	1.3 (0.6, 3.2)	0.52	1.4 (0.6, 3.3)	0.48
	B	IM	0.5 (0.2, 1.2)	0.11	0.7 (0.3, 1.9)	0.47
		MNP	1.1 (0.4, 3.0)	0.79	1.5 (0.6, 4.1)	0.38

HAI: hemagglutination inhibition; FCR: fold change ratio; CI: confidence interval; IM: intramuscular; MNP: microneedle patch;

*Estimated fold change ratio for those in the higher score category compared with those in the lower score category

Table S7. Reaction Severity and Longitudinal HAI GMT.

Reaction	Antigen	Study Group	Unadjusted GMR* (95% CI)	p-value	Adjusted GMR*^ (95% CI)	p-value
Severe (Yes vs. No)	H1N1	IM	1.1 (0.6, 2.2)	0.69	1.1 (0.6, 2.2)	0.75
		MNP	1.3 (0.7, 2.3)	0.42	1.4 (0.8, 2.5)	0.30
	H3N2	IM	0.9 (0.5, 1.6)	0.80	0.8 (0.5, 1.5)	0.52
		MNP	0.8 (0.5, 1.3)	0.41	0.8 (0.5, 1.3)	0.38
	B	IM	1.4 (0.9, 2.0)	0.11	1.1 (0.8, 1.7)	0.58
		MNP	1.1 (0.7, 1.7)	0.77	1.2 (0.8, 1.8)	0.47

HAI: hemagglutination inhibition; GMT: geometric mean titer; GMR: geometric mean titer ratio; CI: confidence interval; IM: intramuscular; MNP: microneedle patch;
*Estimated geometric mean titer ratio for those in the higher severity category compared with those in the lower severity category
^Adjusted for body mass index category, sex, prior inactivated influenza vaccination, race, ethnicity, and study day

Table S8. Reaction Severity and HAI Titer Fold Change by Study Day.

Reaction	Antigen	Study Group	Day 28 FCR* (95% CI)	p-value	Day 180 FCR* (95% CI)	p-value
Unadjusted						
Severe (Yes vs. No)	H1N1	IM	0.7 (0.2, 2.0)	0.46	0.7 (0.2, 2.3)	0.60
		MNP	0.5 (0.2, 1.2)	0.12	0.6 (0.3, 1.5)	0.28
	H3N2	IM	1.7 (0.9, 3.2)	0.12	1.5 (0.8, 2.8)	0.23
		MNP	0.9 (0.5, 1.7)	0.80	1.5 (0.8, 2.7)	0.20
	B	IM	1.0 (0.5, 2.3)	0.91	1.1 (0.5, 2.4)	0.82
		MNP	1.0 (0.5, 2.0)	0.96	1.2 (0.6, 2.3)	0.66
Adjusted^						
Severe (Yes vs. No)	H1N1	IM	0.6 (0.2, 1.9)	0.40	0.7 (0.2, 2.1)	0.53
		MNP	0.6 (0.3, 1.3)	0.17	0.7 (0.3, 1.6)	0.41
	H3N2	IM	1.5 (0.8, 2.8)	0.17	1.4 (0.7, 2.5)	0.32
		MNP	1.0 (0.6, 1.7)	0.99	1.6 (0.9, 2.7)	0.09
	B	IM	1.0 (0.5, 2.1)	0.91	1.1 (0.5, 2.2)	0.80
		MNP	1.1 (0.6, 1.9)	0.87	1.2 (0.7, 2.3)	0.46

HAI: hemagglutination inhibition; FCR: fold change ratio; CI: confidence interval; IM: intramuscular; MNP: microneedle patch;

*Estimated fold change ratio for those in the higher severity category compared with those in the lower severity category

^Adjusted for body mass index category, sex, prior inactivated influenza vaccination, race, ethnicity, and study day

Table S9. Reaction Duration and Longitudinal HAI GMT.

Reaction	Antigen	Study Group	Unadjusted GMR* (95% CI)	p-value	Adjusted GMR*^ (95% CI)	p-value
Prolonged (Yes vs. No)	H1N1	IM	1.0 (0.6, 2.0)	0.90	1.0 (0.5, 2.0)	0.94
		MNP	1.4 (0.8, 2.3)	0.26	1.3 (0.8, 2.3)	0.34
	H3N2	IM	1.2 (0.7, 2.0)	0.58	1.1 (0.6, 1.9)	0.85
		MNP	1.0 (0.6, 1.5)	0.89	1.0 (0.6, 1.6)	1.0
	B	IM	1.3 (0.9, 1.9)	0.18	1.0 (0.7, 1.5)	0.99
		MNP	0.9 (0.6, 1.4)	0.77	1.0 (0.7, 1.5)	0.94

HAI: hemagglutination inhibition; GMT: geometric mean titer; GMR: geometric mean titer ratio; CI: confidence interval; IM: intramuscular; MNP: microneedle patch;

*Estimated geometric mean titer ratio for those in the prolonged category compared with those not in the prolonged category

^Adjusted for body mass index category, sex, prior inactivated influenza vaccination, race, ethnicity, and study day

Table S10. Reaction Duration and HAI Titer Fold Change by Study Day.

Reaction	Antigen	Study Group	Day 28 FCR* (95% CI)	p-value	Day 180 FCR* (95% CI)	p-value
Unadjusted						
Prolonged (Yes vs. No)	H1N1	IM	0.7 (0.2, 2.2)	0.55	0.8 (0.3, 2.5)	0.72
		MNP	0.9 (0.4, 2.0)	0.83	0.8 (0.4, 1.7)	0.58
	H3N2	IM	1.1 (0.6, 2.1)	0.72	1.5 (0.8, 2.9)	0.19
		MNP	1.1 (0.6, 1.8)	0.82	1.4 (0.8, 2.3)	0.25
	B	IM	1.1 (0.5, 2.5)	0.72	1.3 (0.6, 2.8)	0.55
		MNP	0.9 (0.5, 1.7)	0.82	1.0 (0.5, 1.8)	0.92
Adjusted^						
Prolonged (Yes vs. No)	H1N1	IM	0.9 (0.3, 2.8)	0.87	1.0 (0.3, 3.2)	0.93
		MNP	0.7 (0.4, 1.4)	0.33	0.6 (0.3, 1.2)	0.18
	H3N2	IM	1.2 (0.6, 2.2)	0.62	1.6 (0.9, 3.0)	0.14
		MNP	0.9 (0.5, 1.5)	0.66	1.1 (0.7, 1.9)	0.58
	B	IM	1.3 (0.6, 2.6)	0.48	1.4 (0.7, 2.9)	0.34
		MNP	0.6 (0.4, 1.1)	0.08	0.6 (0.4, 1.1)	0.10

HAI: hemagglutination inhibition; FCR: fold change ratio; CI: confidence interval; IM: intramuscular; MNP: microneedle patch;
*Estimated fold change ratio for those in the prolonged category compared with those not in the prolonged category
^Adjusted for body mass index category, sex, prior inactivated influenza vaccination, race, ethnicity, and study day

Table S11. Longitudinal Associations Between Reaction Score and HAI Seroprotection.

Reaction Type	Antigen	Study Group	Unadjusted RR* (95% CI)	p-value	Adjusted RR*^ (95% CI)	p-value
Global	H1N1	IM	1.01 (0.90, 1.12)	0.88	0.99 (0.89, 1.12)	0.92
		MNP	1.03 (0.94, 1.13)	0.55	1.02 (0.93, 1.12)	0.66
	H3N2	IM	0.88 (0.64, 1.20)	0.41	0.87 (0.65, 1.15)	0.33
		MNP	0.94 (0.74, 1.18)	0.58	0.93 (0.75, 1.14)	0.47
	B	IM	1.02 (0.87, 1.19)	0.83	0.96 (0.81, 1.15)	0.67
		MNP	0.98 (0.80, 1.20)	0.83	1.00 (0.82, 1.22)	0.99
Systemic	H1N1	IM	0.92 (0.79, 1.07)	0.28	0.92 (0.80, 1.07)	0.28
		MNP	1.01 (0.86, 1.19)	0.92	0.99 (0.84, 1.15)	0.86
	H3N2	IM	0.75 (0.49, 1.13)	0.16	0.74 (0.51, 1.07)	0.11
		MNP	1.16 (0.96, 1.40)	0.11	1.13 (0.97, 1.33)	0.12
	B	IM	0.99 (0.84, 1.18)	0.94	0.92 (0.78, 1.10)	0.37
		MNP	1.00 (0.69, 1.46)	1.0	0.98 (0.65, 1.48)	0.92
Local	H1N1	IM	0.93 (0.76, 1.14)	0.50	0.91 (0.76, 1.09)	0.32
		MNP	1.08 (1.00, 1.18)	0.06	1.08 (0.98, 1.19)	0.10
	H3N2	IM	0.63 (0.33, 1.21)	0.17	0.61 (0.35, 1.09)	0.10
		MNP	0.84 (0.66, 1.06)	0.15	0.81 (0.65, 1.02)	0.07
	B	IM	0.99 (0.80, 1.24)	0.96	0.95 (0.73, 1.23)	0.70
		MNP	1.00 (0.82, 1.22)	1.0	1.02 (0.84, 1.25)	0.81

HAI: hemagglutination inhibition; RR: risk ratio; CI: confidence interval; IM: intramuscular; MNP: microneedle patch;
*Estimated risk ratio for those in the higher score category compared with those in the lower score category
^Adjusted for body mass index category, sex, prior inactivated influenza vaccination, race, ethnicity, and study day

Table S12. HAI Seroprotection* by Vaccine Route and Day.

Antigen	Study Day	IM (n = 25)	MNP^ (n = 48)	All (n = 73)
H1N1	0	21 (84.0%)	35 (72.9%)	56 (76.7%)
	28	25 (100.0%)	48 (100.0%)	73 (100.0%)
	180	25 (100.0%)	48 (100.0%)	73 (100.0%)
H3N2	0	18 (72.0%)	29 (60.4%)	47 (64.4%)
	28	25 (100.0%)	47 (97.9%)	72 (98.6%)
	180	20 (80.0%)	39 (81.25%)	59 (80.8%)
B	0	19 (76.0%)	23 (47.9%)	42 (57.5%)
	28	25 (100.0%)	47 (97.9%)	72 (98.6%)
	180	23 (92.0%)	38 (79.2%)	61 (83.6%)

*Number (%) of participants with hemagglutination inhibition antibody titer ≥ 1:40
^2 participants had missing outcome data

Table S13A. Longitudinal Associations Between Alternative Cut Point Global Reaction Score and HAI Seroprotection.

Reaction Type	Antigen	Study Group	Unadjusted RR* (95% CI)	p-value	Adjusted RR*^ (95% CI)	p-value
Global (≥ 3 vs ≤ 2)	H1N1	IM	1.01 (0.91, 1.12)	0.79	0.99 (0.88, 1.10)	0.79
		MNP	1.05 (0.96, 1.16)	0.31	1.04 (0.94, 1.15)	0.47
	H3N2	IM	0.98 (0.76, 1.26)	0.89	0.94 (0.73, 1.20)	0.60
		MNP	0.96 (0.79, 1.17)	0.70	0.95 (0.79, 1.14)	0.57
	B	IM	1.09 (0.92, 1.30)	0.30	0.99 (0.83, 1.17)	0.87
		MNP	1.12 (0.90, 1.39)	0.30	1.15 (0.94, 1.41)	0.18
Global (≥ 5 vs ≤ 4)	H1N1	IM	0.98 (0.86, 1.13)	0.80	0.98 (0.85, 1.13)	0.77
		MNP	1.01 (0.91, 1.12)	0.85	1.01 (0.91, 1.12)	0.89
	H3N2	IM	0.85 (0.56, 1.27)	0.42	0.83 (0.58, 1.21)	0.33
		MNP	0.97 (0.76, 1.22)	0.77	0.95 (0.77, 1.18)	0.66
	B	IM	0.96 (0.80, 1.16)	0.69	0.90 (0.75, 1.09)	0.28
		MNP	1.00 (0.82, 1.22)	1.0	1.02 (0.82, 1.26)	0.86

HAI: hemagglutination inhibition; RR: risk ratio; CI: confidence interval; IM: intramuscular; MNP: microneedle patch;
*Estimated risk ratio for those in the higher score category compared with those in the lower score category
^Adjusted for body mass index category, sex, prior inactivated influenza vaccination, race, ethnicity, and study day

Table S13B. Longitudinal Associations Between Alternative Cut Point Systemic Reaction Score and HAI Seroprotection.

Reaction Type	Antigen	Study Group	Unadjusted RR* (95% CI)	p-value	Adjusted RR*^ (95% CI)	p-value
Systemic (≥ 1 vs 0)	H1N1	IM	1.02 (0.92, 1.14)	0.67	1.01 (0.90, 1.13)	0.91
		MNP	1.00 (0.91, 1.10)	0.98	1.00 (0.91, 1.10)	0.99
	H3N2	IM	1.01 (0.78, 1.31)	0.92	0.97 (0.76, 1.24)	0.81
		MNP	1.03 (0.84, 1.25)	0.81	1.03 (0.86, 1.23)	0.78
	B	IM	1.12 (0.93, 1.34)	0.22	1.02 (0.85, 1.21)	0.86
		MNP	1.02 (0.83, 1.25)	0.86	1.05 (0.87, 1.28)	0.59
Systemic (≥ 2 vs ≤ 1)	H1N1	IM	1.00 (0.90, 1.10)	0.93	0.98 (0.88, 1.08)	0.68
		MNP	1.06 (0.97, 1.16)	0.20	1.04 (0.95, 1.13)	0.37
	H3N2	IM	0.92 (0.72, 1.19)	0.54	0.90 (0.71, 1.14)	0.37
		MNP	0.99 (0.80, 1.24)	0.95	0.97 (0.79, 1.19)	0.77
	B	IM	1.12 (0.96, 1.30)	0.16	1.04 (0.89, 1.21)	0.64
		MNP	1.08 (0.88, 1.34)	0.45	1.08 (0.88, 1.31)	0.47

HAI: hemagglutination inhibition; RR: risk ratio; CI: confidence interval; IM: intramuscular; MNP: microneedle patch;
*Estimated risk ratio for those in the higher score category compared with those in the lower score category
^Adjusted for body mass index category, sex, prior inactivated influenza vaccination, race, ethnicity, and study day

Table S13C. Longitudinal Associations Between Alternative Cut Point Local Reaction Score and HAI Seroprotection.

Reaction Type	Antigen	Study Group	Unadjusted RR* (95% CI)	p-value	Adjusted RR*^ (95% CI)	p-value
Local (≥ 1 vs 0)	H1N1	IM	1.05 (0.93, 1.18)	0.45	1.03 (0.91, 1.17)	0.63
		MNP	1.06 (0.89, 1.25)	0.54	1.08 (0.92, 1.27)	0.32
	H3N2	IM	0.94 (0.74, 1.20)	0.63	0.91 (0.71, 1.17)	0.47
		MNP	1.00 (0.73, 1.36)	0.99	1.00 (0.77, 1.29)	0.98
	B	IM	0.96 (0.83, 1.12)	0.62	0.86 (0.73, 1.01)	0.06
		MNP	1.14 (0.76, 1.71)	0.53	1.16 (0.83, 1.62)	0.39
Local (≥ 2 vs ≤ 1)	H1N1	IM	0.98 (0.88, 1.09)	0.67	0.97 (0.87, 1.08)	0.57
		MNP	1.03 (0.93, 1.15)	0.53	1.05 (0.94, 1.17)	0.42
	H3N2	IM	0.99 (0.76, 1.28)	0.92	0.96 (0.75, 1.23)	0.74
		MNP	0.89 (0.74, 1.06)	0.18	0.89 (0.74, 1.07)	0.21
	B	IM	1.01 (0.87, 1.18)	0.87	0.92 (0.78, 1.09)	0.35
		MNP	1.13 (0.87, 1.47)	0.37	1.15 (0.88, 1.51)	0.31

HAI: hemagglutination inhibition; RR: risk ratio; CI: confidence interval; IM: intramuscular; MNP: microneedle patch;
*Estimated risk ratio for those in the higher score category compared with those in the lower score category
^Adjusted for body mass index category, sex, prior inactivated influenza vaccination, race, ethnicity, and study day

Table S14. Longitudinal Associations Between Reaction Score and HAI Seroconversion.

Reaction Type	Antigen	Study Group	Unadjusted RR* (95% CI)	p-value	Adjusted RR*^ (95% CI)	p-value
Global	H1N1	IM	1.05 (0.59, 1.87)	0.86	1.05 (0.62, 1.78)	0.85
		MNP	0.96 (0.75, 1.24)	0.77	0.98 (0.78, 1.24)	0.86
	H3N2	IM	1.65 (0.99, 2.76)	0.05	1.59 (0.96, 2.63)	0.07
		MNP	0.97 (0.65, 1.45)	0.89	0.97 (0.71, 1.33)	0.85
	B	IM	1.29 (0.37, 4.41)	0.69	1.20 (0.43, 3.31)	0.73
		MNP	1.53 (0.96, 2.43)	0.07	1.38 (0.89, 2.14)	0.15
Systemic	H1N1	IM	1.10 (0.59, 2.05)	0.76	1.06 (0.59, 1.91)	0.84
		MNP	0.97 (0.69, 1.37)	0.87	1.04 (0.71, 1.52)	0.83
	H3N2	IM	**1.69 (1.08, 2.65)**	**0.02**	1.53 (0.91, 2.58)	0.11
		MNP	1.25 (0.64, 2.45)	0.52	**1.50 (1.21, 1.85)**	**<0.01**
	B	IM	2.26 (0.69, 7.39)	0.18	1.83 (0.70, 4.79)	0.22
		MNP	1.10 (0.53, 2.30)	0.80	1.26 (0.72, 2.20)	0.41
Local	H1N1	IM	0.51 (0.10, 2.54)	0.41	0.52 (0.11, 2.53)	0.41
		MNP	1.02 (0.81, 1.29)	0.87	1.00 (0.79, 1.26)	0.99
	H3N2	IM	1.54 (0.94, 2.55)	0.09	1.57 (0.88, 2.80)	0.13
		MNP	1.16 (0.82, 1.64)	0.40	1.06 (0.80, 1.40)	0.70
	B	IM	1.47 (0.26, 8.29)	0.66	1.35 (0.28, 6.55)	0.71
		MNP	1.52 (0.95, 2.43)	0.08	1.14 (0.69, 1.90)	0.61

HAI: hemagglutination inhibition; RR: risk ratio; CI: confidence interval; IM: intramuscular; MNP: microneedle patch;
*Estimated risk ratio for those in the higher score category compared with those in the lower score category
^Adjusted for body mass index category, sex, prior inactivated influenza vaccination, race, ethnicity, and study day

Table S15A. Reaction Score and H1N1 HAI Seroconversion by Study Day.

Reaction Type	Study Group	Day 28 RR* (95% CI)	p-value	Day 180 RR* (95% CI)	p-value
Unadjusted					
Global	IM	0.9 (0.5, 1.4)	0.56	1.5 (0.6, 3.5)	0.38
	MNP	1 (0.8, 1.2)	0.87	0.9 (0.5, 1.6)	0.77
Systemic	IM	0.8 (0.4, 1.4)	0.44	1.8 (0.8, 4.1)	0.15
	MNP	1.1 (1.0, 1.2)	0.08	0.8 (0.3, 2.2)	0.69
Local	IM	0.4 (0.1, 1.9)	0.25	0.7 (0.1, 3.9)	0.71
	MNP	1.0 (0.9, 1.2)	1.0	1.1 (0.7, 1.7)	0.83
Adjusted^					
Global	IM	0.9 (0.5, 1.4)	0.59	1.5 (0.7, 3.3)	0.33
	MNP	1.0 (0.8, 1.2)	0.98	0.9 (0.6, 1.5)	0.81
Systemic	IM	0.8 (0.4, 1.4)	0.41	1.8 (0.8, 3.9)	0.14
	MNP	1.1 (0.9, 1.4)	0.18	0.9 (0.3, 2.4)	0.78
Local	IM	0.4 (0.1, 2.0)	0.26	0.8 (0.2, 3.9)	0.75
	MNP	1.0 (0.8, 1.2)	0.84	1.0 (0.7, 1.6)	0.89

HAI: hemagglutination inhibition; RR: risk ratio; CI: confidence interval; IM: intramuscular; MNP: microneedle patch;
*Estimated risk ratio for those in the higher score category compared with those in the lower score category
^Adjusted for body mass index category, sex, prior inactivated influenza vaccination, race, ethnicity, and study day

Table S15B. Reaction Score and H3N2 HAI Seroconversion by Study Day.

Reaction Type	Study Group	Day 28 RR* (95% CI)	p-value	Day 180 RR* (95% CI)	p-value
Unadjusted					
Global	IM	1.2 (0.8, 1.8)	0.42	7.7 (1.0, 62.2)	0.06
Global	MNP	0.9 (0.6, 1.3)	0.45	1.4 (0.5, 4.0)	0.54
Systemic	IM	**1.5 (1.1, 2.0)**	**0.01**	3.2 (0.6, 17.9)	0.19
Systemic	MNP	0.9 (0.5, 1.7)	0.84	2.4 (0.8, 7.6)	0.12
Local	IM	**1.4 (1.1, 1.8)**	**0.01**	2.4 (0.4, 16.6)	0.36
Local	MNP	1 (0.8, 1.4)	0.8	1.7 (0.6, 4.6)	0.33
Adjusted^					
Global	IM	1.1 (0.8, 1.7)	0.56	7.3 (0.9, 56.5)	0.06
Global	MNP	0.9 (0.6, 1.2)	0.37	1.4 (0.5, 3.6)	0.52
Systemic	IM	1.3 (0.9, 1.8)	0.11	2.8 (0.5, 16.5)	0.24
Systemic	MNP	1.1 (0.8, 1.6)	0.57	**2.9 (1.4, 6.0)**	**<0.01**
Local	IM	**1.4 (1.0, 1.9)**	**0.03**	2.5 (0.3, 17.8)	0.37
Local	MNP	0.9 (0.7, 1.3)	0.71	1.5 (0.6, 3.8)	0.38

HAI: hemagglutination inhibition; RR: risk ratio; CI: confidence interval; IM: intramuscular; MNP: microneedle patch;
*Estimated risk ratio for those in the higher score category compared with those in the lower score category
^Adjusted for body mass index category, sex, prior inactivated influenza vaccination, race, ethnicity, and study day

Table S15C. Reaction Score and B HAI Seroconversion by Study Day.

Reaction Type	Study Group	Day 28 RR* (95% CI)	p-value	Day 180 RR* (95% CI)	p-value
Unadjusted					
Global	IM	1.5 (0.5, 4.8)	0.45	0.9 (0.1, 6.9)	0.88
	MNP	1.3 (0.9, 2.0)	0.15	2.1 (0.9, 5.1)	0.11
Systemic	IM	1.9 (0.6, 5.7)	0.25	3.2 (0.6, 17.9)	0.19
	MNP	1.2 (0.6, 2.2)	0.60	0.9 (0.2, 5.4)	0.92
Local	IM	1.0 (0.2, 5.8)	0.96	2.4 (0.4, 16.6)	0.36
	MNP	1.3 (0.8, 1.9)	0.26	2.3 (0.9, 5.8)	0.07
Adjusted^					
Global	IM	1.3 (0.5, 3.3)	0.52	0.7 (0.1, 6.3)	0.79
	MNP	1.3 (0.8, 2.0)	0.32	2.0 (0.9, 4.4)	0.11
Systemic	IM	1.6 (0.7, 3.9)	0.26	2.7 (0.5, 15.1)	0.25
	MNP	1.3 (0.7, 2.7)	0.43	1.0 (0.3, 3.6)	0.96
Local	IM	1.1 (0.2, 5.1)	0.92	2.5 (0.4, 15.6)	0.32
	MNP	1.0 (0.6, 1.7)	0.96	1.9 (0.7, 4.7)	0.18

HAI: hemagglutination inhibition; RR: risk ratio; CI: confidence interval; IM: intramuscular; MNP: microneedle patch;
*Estimated risk ratio for those in the higher score category compared with those in the lower score category
^Adjusted for body mass index category, sex, prior inactivated influenza vaccination, race, ethnicity, and study day

Table S16A. Longitudinal Associations Between Alternative Cut Point Global Reaction Score and HAI Seroconversion.

Reaction Type	Antigen	Study Group	Unadjusted RR* (95% CI)	p-value	Adjusted RR*^ (95% CI)	p-value
Global (≥ 3 vs ≤ 2)	H1N1	IM	1.09 (0.67, 1.76)	0.73	1.21 (0.76, 1.93)	0.43
		MNP	0.84 (0.68, 1.04)	0.11	0.86 (0.69, 1.06)	0.16
	H3N2	IM	1.02 (0.61, 1.72)	0.94	0.98 (0.59, 1.64)	0.95
		MNP	0.88 (0.62, 1.24)	0.46	0.87 (0.64, 1.18)	0.36
	B	IM	1.57 (0.43, 5.69)	0.49	1.61 (0.53, 4.88)	0.40
		MNP	0.94 (0.58, 1.52)	0.80	0.84 (0.53, 1.32)	0.45
Global (≥ 5 vs ≤ 4)	H1N1	IM	0.96 (0.45, 2.05)	0.92	0.97 (0.47, 1.98)	0.93
		MNP	1.04 (0.80, 1.35)	0.79	1.02 (0.79, 1.31)	0.87
	H3N2	IM	**1.75 (1.12, 2.74)**	**0.01**	1.63 (0.93, 2.86)	0.09
		MNP	1.08 (0.73, 1.60)	0.69	1.04 (0.77, 1.41)	0.78
	B	IM	1.33 (0.34, 5.26)	0.68	1.21 (0.38, 3.81)	0.74
		MNP	1.71 (1.10, 2.68)	0.02	1.42 (0.91, 2.22)	0.13

HAI: hemagglutination inhibition; RR: risk ratio; CI: confidence interval; IM: intramuscular; MNP: microneedle patch;
*Estimated risk ratio for those in the higher score category compared with those in the lower score category
^Adjusted for body mass index category, sex, prior inactivated influenza vaccination, race, ethnicity, and study day

Table S16B. Longitudinal Associations Between Alternative Cut Point Systemic Reaction Score and HAI Seroconversion.

Reaction Type	Antigen	Study Group	Unadjusted RR* (95% CI)	p-value	Adjusted RR*^ (95% CI)	p-value
Systemic (≥ 1 vs 0)	H1N1	IM	1.06 (0.64, 1.73)	0.83	1.15 (0.71, 1.87)	0.57
		MNP	0.94 (0.75, 1.18)	0.60	0.95 (0.77, 1.16)	0.60
	H3N2	IM	1.04 (0.61, 1.75)	0.89	1.02 (0.61, 1.71)	0.93
		MNP	1.04 (0.73, 1.47)	0.83	1.03 (0.77, 1.37)	0.84
	B	IM	2.00 (0.45, 8.83)	0.36	2.16 (0.58, 8.05)	0.25
		MNP	0.93 (0.57, 1.50)	0.76	0.86 (0.58, 1.29)	0.47
Systemic (≥ 2 vs ≤ 1)	H1N1	IM	0.89 (0.55, 1.46)	0.65	0.95 (0.60, 1.50)	0.81
		MNP	0.93 (0.71, 1.22)	0.60	1.04 (0.80, 1.35)	0.79
	H3N2	IM	0.99 (0.58, 1.71)	0.98	1.06 (0.65, 1.74)	0.81
		MNP	0.87 (0.58, 1.32)	0.52	0.98 (0.71, 1.35)	0.90
	B	IM	1.52 (0.42, 5.44)	0.52	1.8 (0.62, 5.23)	0.28
		MNP	1.01 (0.60, 1.70)	0.97	1.38 (0.87, 2.20)	0.17

HAI: hemagglutination inhibition; RR: risk ratio; CI: confidence interval; IM: intramuscular; MNP: microneedle patch;
*Estimated risk ratio for those in the higher score category compared with those in the lower score category
^Adjusted for body mass index category, sex, prior inactivated influenza vaccination, race, ethnicity, and study day

Table S16C. Longitudinal Associations Between Alternative Cut Point Local Reaction Score and HAI Seroconversion.

Reaction Type	Antigen	Study Group	Unadjusted RR* (95% CI)	p-value	Adjusted RR*^ (95% CI)	p-value
Local (≥ 1 vs 0)	H1N1	IM	0.75 (0.49, 1.14)	0.17	0.74 (0.50, 1.11)	0.15
		MNP	0.96 (0.61, 1.51)	0.86	0.87 (0.56, 1.34)	0.52
	H3N2	IM	0.88 (0.53, 1.46)	0.63	1.02 (0.65, 1.60)	0.95
		MNP	1.02 (0.57, 1.83)	0.94	0.93 (0.61, 1.44)	0.76
	B	IM	0.94 (0.27, 3.30)	0.92	1.01 (0.37, 2.77)	0.98
		MNP	0.74 (0.49, 1.10)	0.14	**0.42 (0.24, 0.72)**	**<0.01**
Local (≥ 2 vs ≤ 1)	H1N1	IM	1.24 (0.77, 1.99)	0.39	1.28 (0.81, 2.03)	0.30
		MNP	1.07 (0.82, 1.41)	0.61	0.98 (0.77, 1.25)	0.88
	H3N2	IM	1.64 (0.99, 2.71)	0.05	1.54 (0.97, 2.46)	0.07
		MNP	0.94 (0.66, 1.33)	0.72	0.87 (0.64, 1.17)	0.35
	B	IM	2.10 (0.63, 7.01)	0.23	1.75 (0.65, 4.69)	0.27
		MNP	1.36 (0.83, 2.23)	0.22	0.81 (0.47, 1.40)	0.44

HAI: hemagglutination inhibition; RR: risk ratio; CI: confidence interval; IM: intramuscular; MNP: microneedle patch;
*Estimated risk ratio for those in the higher score category compared with those in the lower score category
^Adjusted for body mass index category, sex, prior inactivated influenza vaccination, race, ethnicity, and study day

Table S17A. Alternative Cut Point Global Reaction Score and HAI Seroconversion by Study Day.

Reaction Type	Antigen	Study Group	Day 28 RR* (95% CI)	p-value	Day 180 RR* (95% CI)	p-value
Unadjusted						
Global (≥ 3 vs ≤ 2)	H1N1	IM	1.0 (0.6, 1.4)	0.84	1.4 (0.5, 3.5)	0.51
		MNP	1.0 (0.8, 1.1)	0.76	0.7 (0.4, 1.0)	0.08
	H3N2	IM	0.9 (0.6, 1.3)	0.54	2.4 (0.3, 19.7)	0.43
		MNP	0.9 (0.7, 1.2)	0.38	0.9 (0.3, 2.4)	0.77
	B	IM	1.3 (0.4, 4.3)	0.66	2.4 (0.3, 19.7)	0.43
		MNP	0.9 (0.6, 1.3)	0.50	1.1 (0.4, 3.0)	0.78
Adjusted^						
Global (≥ 3 vs ≤ 2)	H1N1	IM	1.1 (0.7, 1.7)	0.74	1.5 (0.6, 3.7)	0.34
		MNP	1.0 (0.8, 1.2)	0.87	0.7 (0.4, 1.0)	0.07
	H3N2	IM	0.8 (0.5, 1.3)	0.43	2.3 (0.3, 18.5)	0.44
		MNP	0.9 (0.6, 1.2)	0.37	0.8 (0.3, 2.2)	0.74
	B	IM	1.4 (0.5, 4.1)	0.49	2.6 (0.3, 21.5)	0.38
		MNP	0.8 (0.5, 1.3)	0.33	1.0 (0.4, 2.5)	0.92
Unadjusted						
Global (≥ 5 vs ≤ 4)	H1N1	IM	0.7 (0.3, 1.5)	0.36	1.5 (0.6, 3.7)	0.37
		MNP	1 (0.8, 1.2)	0.76	1.1 (0.7, 1.9)	0.59
	H3N2	IM	**1.4 (1.1, 1.9)**	**0.01**	4.0 (0.7, 21.8)	0.11
		MNP	0.9 (0.6, 1.3)	0.70	1.7 (0.6, 4.9)	0.31
	B	IM	1.3 (0.4, 4.7)	0.66	1.3 (0.2, 10.3)	0.78
		MNP	1.4 (1.0, 2.1)	0.06	**2.6 (1.1, 6.2)**	**0.03**
Adjusted^						
Global (≥ 5 vs ≤ 4)	H1N1	IM	0.7 (0.4, 1.5)	0.38	1.5 (0.7, 3.6)	0.31
		MNP	1.0 (0.8, 1.2)	0.72	1.1 (0.7, 1.8)	0.60
	H3N2	IM	1.3 (0.9, 1.8)	0.11	3.7 (0.6, 21.2)	0.14
		MNP	0.9 (0.6, 1.2)	0.49	1.6 (0.6, 4.2)	0.31
	B	IM	1.2 (0.4, 3.3)	0.72	1.2 (0.2, 9.3)	0.86
		MNP	1.2 (0.8, 2.0)	0.34	2.2 (1.0, 5.1)	0.05

HAI: hemagglutination inhibition; RR: risk ratio; CI: confidence interval; IM: intramuscular; MNP: microneedle patch;

*Estimated risk ratio for those in the higher score category compared with those in the lower score category

^Adjusted for body mass index category, sex, prior inactivated influenza vaccination, race, ethnicity, and study day

Table S17B. Alternative Cut Point Systemic Reaction Score and HAI Seroconversion by Study Day.

Reaction Type	Antigen	Study Group	Day 28 RR* (95% CI)	p-value	Day 180 RR* (95% CI)	p-value
Unadjusted						
Systemic (≥ 1 vs 0)	H1N1	IM	1.0 (0.7, 1.5)	1.0	1.2 (0.5, 3.0)	0.75
		MNP	1.0 (0.8, 1.1)	0.65	0.9 (0.6, 1.4)	0.67
	H3N2	IM	0.9 (0.6, 1.4)	0.69	2 (0.2, 16.6)	0.52
		MNP	0.9 (0.7, 1.3)	0.67	1.5 (0.5, 4.4)	0.48
	B	IM	2.0 (0.5, 8.0)	0.33	2.0 (0.2, 16.6)	0.52
		MNP	0.9 (0.6, 1.4)	0.63	1.0 (0.4, 2.5)	0.98
Adjusted^						
Systemic (≥ 1 vs 0)	H1N1	IM	1.1 (0.7, 1.7)	0.69	1.3 (0.5, 3.1)	0.59
		MNP	1.0 (0.8, 1.1)	0.71	0.9 (0.6, 1.4)	0.66
	H3N2	IM	0.9 (0.6, 1.4)	0.66	2.0 (0.2, 16.0)	0.52
		MNP	0.9 (0.7, 1.2)	0.63	1.5 (0.5, 3.9)	0.45
	B	IM	2.2 (0.6, 7.6)	0.23	2.2 (0.3, 17.7)	0.48
		MNP	0.9 (0.6, 1.3)	0.44	0.9 (0.4, 2.1)	0.86
Unadjusted						
Systemic (≥ 2 vs ≤ 1)	H1N1	IM	0.9 (0.6, 1.3)	0.56	0.9 (0.4, 2.2)	0.82
		MNP	1.0 (0.8, 1.2)	0.81	0.9 (0.5, 1.5)	0.59
	H3N2	IM	0.8 (0.5, 1.3)	0.31	3.2 (0.4, 27.2)	0.28
		MNP	1.0 (0.7, 1.4)	0.82	0.6 (0.1, 2.4)	0.47
	B	IM	1.8 (0.5, 6.0)	0.33	1.1 (0.2, 6.5)	0.93
		MNP	1.1 (0.7, 1.7)	0.67	0.8 (0.3, 2.5)	0.71
Adjusted^						
Systemic (≥ 2 vs ≤ 1)	H1N1	IM	0.9 (0.6, 1.4)	0.78	1.0 (0.4, 2.2)	0.92
		MNP	1.1 (0.9, 1.3)	0.42	0.9 (0.5, 1.7)	0.86
	H3N2	IM	0.8 (0.5, 1.3)	0.42	3.5 (0.4, 27.5)	0.24
		MNP	1.1 (0.8, 1.5)	0.63	0.7 (0.2, 2.4)	0.54
	B	IM	2.0 (0.7, 5.5)	0.18	1.2 (0.2, 6.9)	0.84
		MNP	1.5 (0.9, 2.3)	0.12	1.1 (0.4, 3.0)	0.89

HAI: hemagglutination inhibition; RR: risk ratio; CI: confidence interval; IM: intramuscular; MNP: microneedle patch;

*Estimated risk ratio for those in the higher score category compared with those in the lower score category

^Adjusted for body mass index category, sex, prior inactivated influenza vaccination, race, ethnicity, and study day

Table S17C. Alternative Cut Point Local Reaction Score and HAI Seroconversion by Study Day.

Reaction Type	Antigen	Study Group	Day 28 RR* (95% CI)	p-value	Day 180 RR* (95% CI)	p-value
Unadjusted						
Local (≥ 1 vs 0)	H1N1	IM	**0.7 (0.5, 1.0)**	**0.03**	0.8 (0.3, 2.0)	0.67
		MNP	1.2 (0.8, 1.9)	0.44	0.7 (0.4, 1.2)	0.22
	H3N2	IM	0.8 (0.5, 1.2)	0.30	1.4 (0.2, 11.5)	0.75
		MNP	1.0 (0.6, 1.6)	0.96	1.2 (0.2, 7.3)	0.87
	B	IM	0.8 (0.2, 2.5)	0.68	1.4 (0.2, 11.5)	0.75
		MNP	**0.6 (0.5, 0.8)**	**<0.01**	1.4 (0.2, 8.6)	0.72
Adjusted^						
Local (≥ 1 vs 0)	H1N1	IM	0.7 (0.5, 1.0)	0.06	0.8 (0.4, 1.9)	0.66
		MNP	1.1 (0.7, 1.6)	0.78	0.6 (0.4, 1.1)	0.08
	H3N2	IM	0.9 (0.6, 1.4)	0.70	1.6 (0.2, 12.8)	0.64
		MNP	0.9 (0.6, 1.4)	0.66	1.1 (0.2, 5.4)	0.94
	B	IM	0.9 (0.4, 2.3)	0.86	1.7 (0.2, 13.9)	0.64
		MNP	**0.4 (0.2, 0.6)**	**<0.01**	0.8 (0.1, 5.8)	0.86
Unadjusted						
Local (≥ 2 vs ≤ 1)	H1N1	IM	1.0 (0.7, 1.5)	1.0	1.8 (0.7, 4.3)	0.19
		MNP	1.1 (0.9, 1.4)	0.29	1.0 (0.6, 1.7)	0.97
	H3N2	IM	1.3 (0.9, 2.0)	0.15	4.5 (0.5, 37.4)	0.16
		MNP	0.9 (0.7, 1.2)	0.34	1.2 (0.4, 3.9)	0.75
	B	IM	1.5 (0.5, 4.7)	0.48	4.5 (0.5, 37.4)	0.16
		MNP	1.0 (0.6, 1.5)	0.84	5.5 (0.8, 38.2)	0.09
Adjusted^						
Local (≥ 2 vs ≤ 1)	H1N1	IM	1.0 (0.7, 1.6)	0.83	1.9 (0.8, 4.3)	0.13
		MNP	1.0 (0.8, 1.2)	0.81	0.9 (0.6, 1.5)	0.75
	H3N2	IM	1.3 (0.9, 1.9)	0.24	4.2 (0.5, 33.3)	0.17
		MNP	0.8 (0.6, 1.1)	0.11	1.1 (0.4, 3.4)	0.84
	B	IM	1.4 (0.6, 3.4)	0.45	4.2 (0.5, 35.8)	0.19
		MNP	0.7 (0.4, 1.1)	0.13	3.8 (0.5, 28.1)	0.20

HAI: hemagglutination inhibition; RR: risk ratio; CI: confidence interval; IM: intramuscular; MNP: microneedle patch;
*Estimated risk ratio for those in the higher score category compared with those in the lower score category

^Adjusted for body mass index category, sex, prior inactivated influenza vaccination, race, ethnicity, and study day

Supplemental Figures

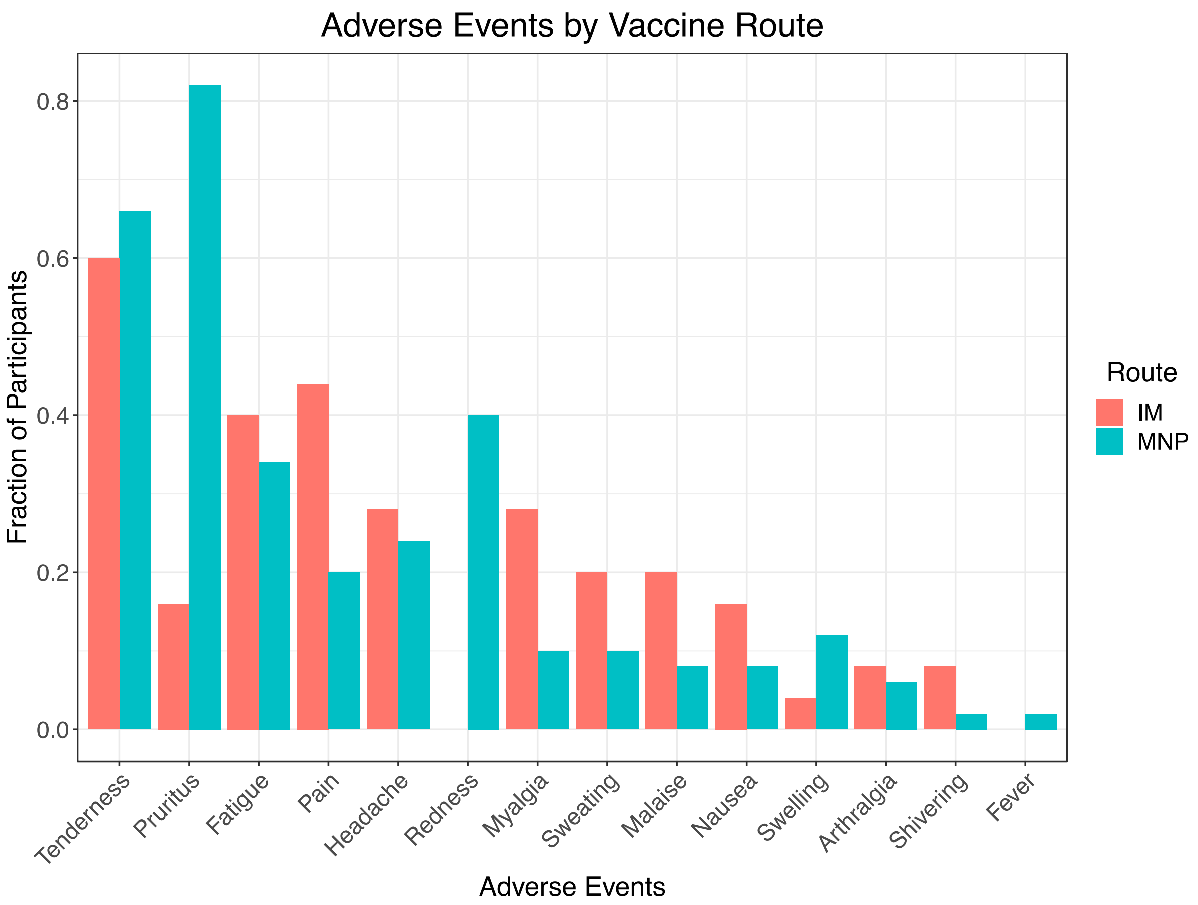

Figure S1. Adverse Events by Vaccine Route. Bar graph of the fraction of participants in the intramuscular (IM, red) and microneedle patch (MNP, teal) group who developed each type of adverse event. Fraction refers to the number of participants with a given adverse event in each group divided by the number of participants in the group. Adverse events are ordered on the X-axis by cumulative number of participants who reported the event.

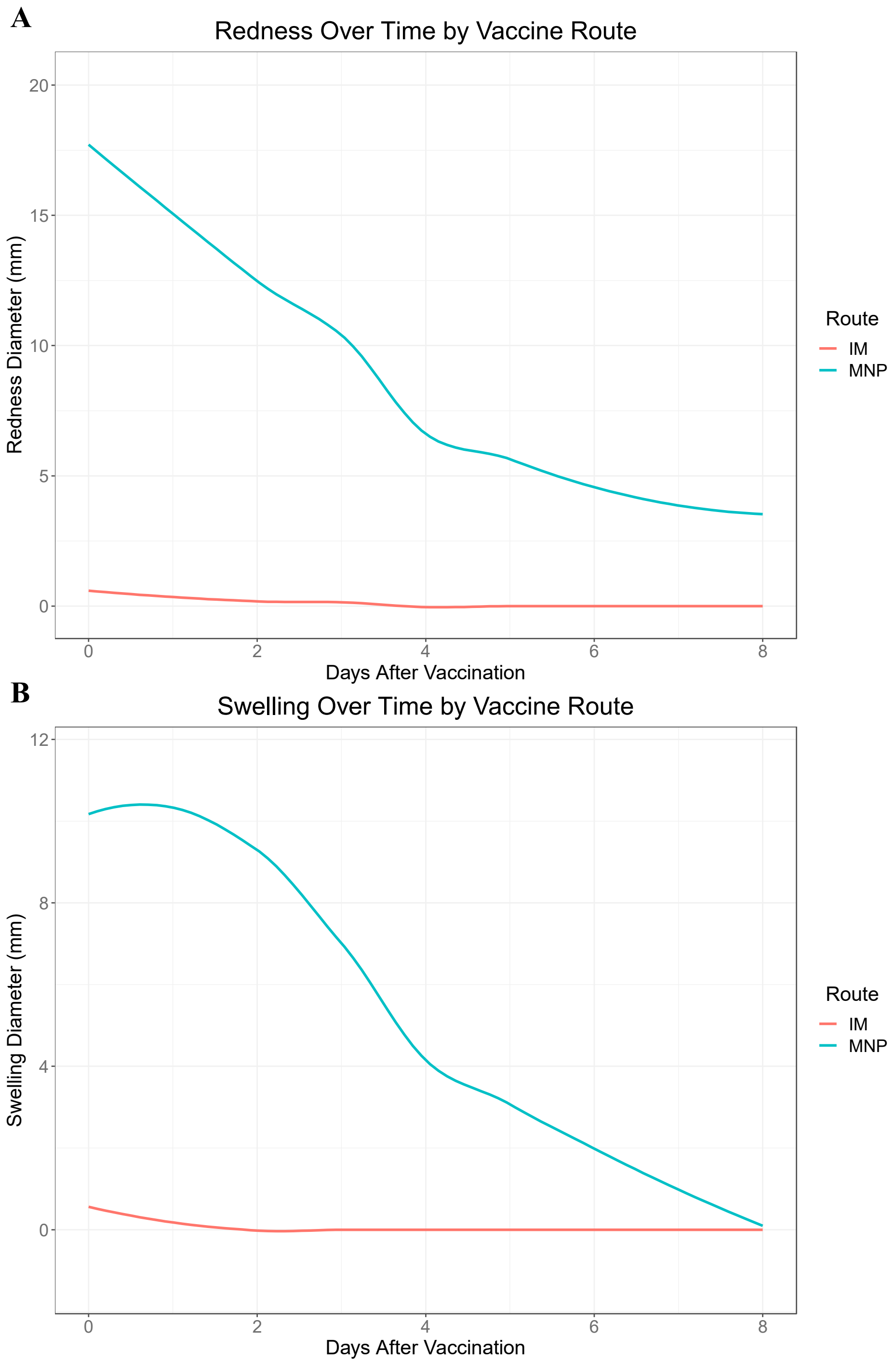

Figure S2. Select Local Adverse Events by Time and Vaccine Route. Smoothed conditional means graphs of redness (A) and swelling (B) diameter in millimeters over time after vaccination for those in the intramuscular (IM, red) and microneedle patch (MNP, teal) groups.

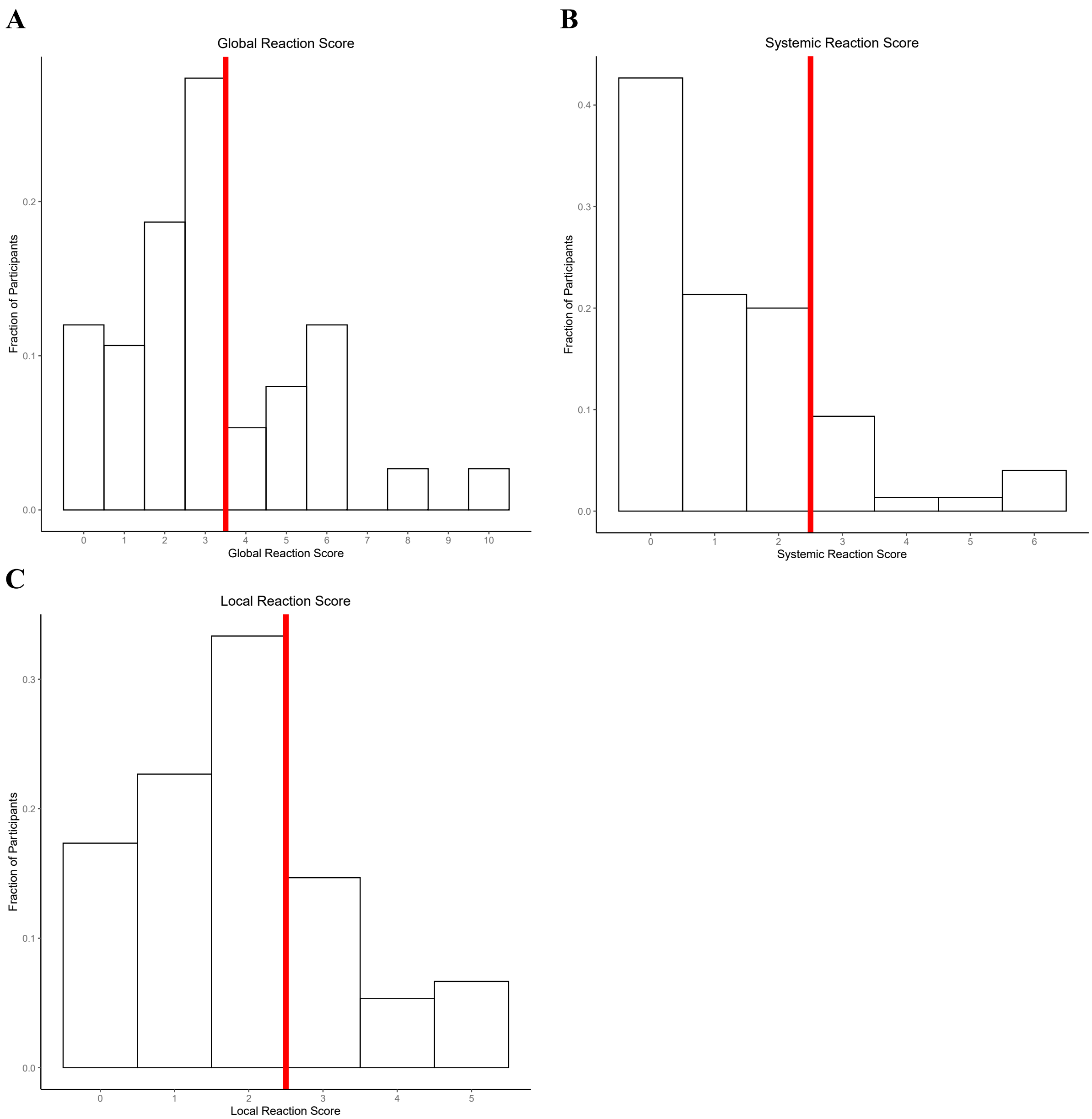

Figure S3. Reaction Score Univariable Distributions. Histograms of the number of participants who scored at each level of the Global (A), Systemic (B), and Local (C) reaction scores. Vertical red lines depict the dichotomization cut points chosen for the primary analyses.

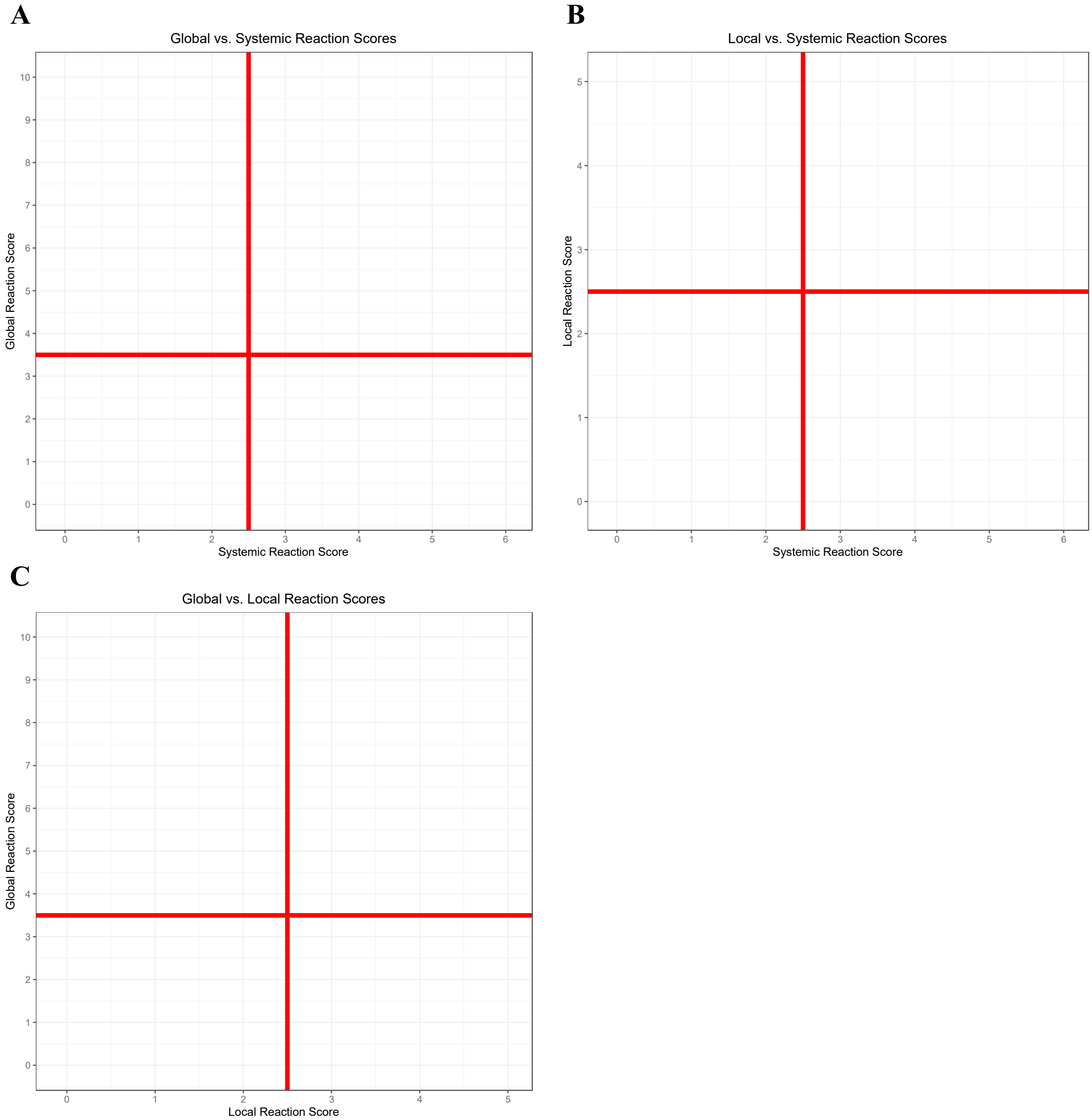

Figure S4. Reaction Score Bivariable Distributions. Scatterplots of the Global and Systemic (A), Local and Systemic (B), and Global and Local (C) reaction scores. Red lines depict the dichotomization cut points chosen for the primary analyses. Each point represents an individual participant.

A

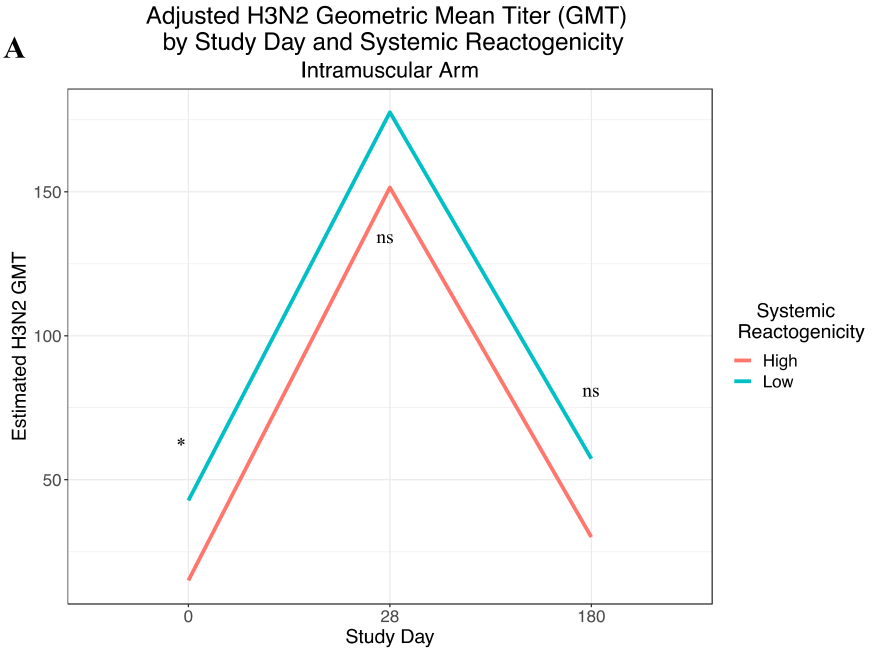

B

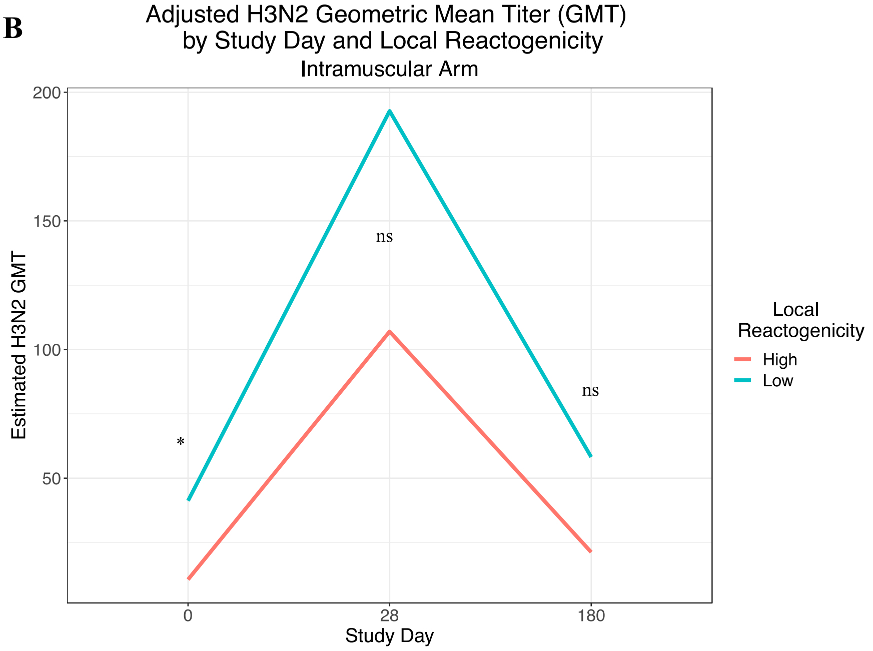

Figure S5. H3N2 HAI Titer by Time and Reaction Score. Estimated adjusted geometric mean titer against the H3N2 antigen of intramuscular seasonal inactivated influenza vaccine by study day, comparing low and high score levels for systemic reactogenicity (A) and local reactogenicity (B). * denotes p = 0.01 and ns denotes a comparison that is not statistically significant between the low and high score levels at the indicated time point.
